# Phosphorylation of the AMPA receptor GluA1 subunit regulates memory load capacity

**DOI:** 10.1007/s00429-014-0927-1

**Published:** 2014-11-08

**Authors:** Laura Olivito, Paola Saccone, Valentina Perri, Julia L. Bachman, Paola Fragapane, Andrea Mele, Richard L. Huganir, Elvira De Leonibus

**Affiliations:** 1 Institute of Genetics and Biophysics, CNR, Via P. Castellino 111, 80131 Naples, Italy; 2 Telethon Institute of Genetics and Medicine (TIGEM), Naples, Italy; 3 Dipartimento di Biologia e Biotecnologie, Università degli Studi di Roma “La Sapienza”, Rome, Italy; 4 Centro di Ricerca in Neurobiologia-D. Bovet, Università degli Studi di Roma “La Sapienza”, Rome, Italy; 5 Department of Neuroscience and Howard Hughes Medical Institute, Johns Hopkins University School of Medicine, Hunterian 1001, 725 North Wolfe Street, Baltimore, MD 21205 USA; 6 Istituto di Biologia e Patologia Molecolare, CNR, Rome, Italy

**Keywords:** AMPA-receptors phosphorylation, Hippocampus, Working memory capacity, Long-term memory, Protein kinases

## Abstract

Memory capacity (MC) refers to the number of elements one can maintain for a short retention interval. The molecular mechanisms underlying MC are unexplored. We have recently reported that mice as well as humans have a limited MC, which is reduced by hippocampal lesions. Here, we addressed the molecular mechanisms supporting MC. GluA1 AMPA-receptors (AMPA-R) mediate the majority of fast excitatory synaptic transmission in the brain and are critically involved in memory. Phosphorylation of GluA1 at serine residues S831 and S845 is promoted by CaMKII and PKA, respectively, and regulates AMPA-R function in memory duration. We hypothesized that AMPA-R phosphorylation may also be a key plastic process for supporting MC because it occurs in a few minutes, and potentiates AMPA-R ion channel function. Here, we show that knock-in mutant mice that specifically lack both of S845 and S831 phosphorylation sites on the GluA1 subunit had reduced MC in two different behavioral tasks specifically designed to assess MC in mice. This demonstrated *a causal link* between AMPA-R phosphorylation and MC. We then showed that information load *regulates* AMPA-R phosphorylation within the hippocampus, and that an overload condition associated with impaired memory is paralleled by a lack of AMPA-R phosphorylation. Accordingly, we showed that in conditions of high load, but not of low load, the pharmacological inhibition of the NMDA–CaMKII–PKA pathways within the hippocampus prevents memory as well as associated AMPA-R phosphorylation. These data provide the first identified molecular mechanism that regulates MC.

**Electronic supplementary material:**

The online version of this article (doi:10.1007/s00429-014-0927-1) contains supplementary material, which is available to authorized users.

## Introduction

Memory capacity (MC) refers to the limited capacity of working memory (WM). George Miller suggested that humans are capable of holding seven, plus or minus two digits, of information in a short-retention time interval (RTI); this limit is called memory span. MC is necessary for complex reasoning and multitasking in humans (Engle et al. 1999). MC deficits have been shown to predict the severity of dementia in Alzheimer’ disease and are a core symptom of schizophrenia (Saunders and Summers 2010). Nevertheless, there are few studies about the neurobiology of MC.

MC has been generally associated with frontostriatal dopamine (DA) activity in humans (Klostermann et al. 2012; Cools et al. 2008), and with acetylcholine receptors activation in rodents (Tarantino et al. 2011; Young et al. 2011). Recently, the attention has shifted to the role of the hippocampus in mediating MC. Patients and monkeys with hippocampal damage and amnesic patients have reduced item, digit or spatial memory at short RTI (Shrager et al. 2008; Beason-Held et al. 1999; Levy et al. 2004). We have recently confirmed these results through a novel behavioral procedure, the Different-Objects-Task/Identical-Object-Task (DOT–IOT), developed to study MC in rodents (Sannino et al. 2012). Using the DOT–IOT we showed that outbred CD1 mice, as well as humans, have limited object MC at short RTI (1 min), as they can discriminate up to 6, but not 9, different objects. Dorsal hippocampal lesions reduced object MC from 6 to 4 (Sannino et al. 2012). These results suggest that the hippocampus might be a possible neural substrate that is activated *to process high information load*.

The observed role of the hippocampus in MC raises an interesting issue regarding the molecular mechanisms required to process high information load, which has never been explored before. Glutamate gated ion channels including *N*-methyl-d-aspartate receptor (NMDA-R) and GluA1 a-amino-3-hydroxy-5-methyl-4-isoxazolepropionic acid (AMPA-R) are crucial for synaptic plasticity and memory formation. Memory at short RTI involves a rapid, albeit transient, induction of post-translational modifications of substrate proteins (Dash et al. 2007), such as protein phosphorylation. AMPA-Rs mediate excitatory synaptic transmission, synaptic plasticity and memory (Zamanillo et al. 1999; Reisel et al. 2002; Sanderson et al. 2009). Phosphorylation of GluA1 at serine residues S831 and S845 regulates AMPA-R function through two mechanisms: modulation of ion channel properties and regulation of the synaptic targeting of the receptor (Benke et al. 1998; Hayashi et al. 2000; Shi et al. 1999, 2001). Therefore, these phosphorylation events are interesting because they can occur at short RTI and potentiate AMPA receptor ion channel function (Barria et al. 1997a, b; Derkach et al. 1999; Banke et al. 2000). These sites may also be a key process for regulating MC. A specific role of AMPA-R phosphorylation in the stabilization of LTM has been previously demonstrated (Ferretti et al. 2014; Lee et al. 2003); however, their role in WM has been never proved before.

In this study, by modulating the memory load in two different behavioral tasks for mice, the radial maze and the DOT/IOT, we demonstrate that AMPA-R phosphorylation regulates and is regulated by memory load capacity.

## Materials and methods

### Subjects

All the biochemical and pharmacological experiments were performed in outbred CD1 adult (10–16 weeks) male mice (Charles River, Italy, RRID: rid_000091), because the DOT–IOT task was originally set up for this strain (Sannino et al. 2012). Then we switched to mice with serine-to-alanine mutations of GluA1 S831 and S845 phosphorylation sites (referred here-after as phospho-free mice), which were generated and genotyped after birth as previously described (Lee et al. 2003). Mutation sites were verified using phosphorylation-selective antibodies against GluA1. Wild type (WT) and homozygous (phospho-free) male mice, aged 14–21 weeks, with C57BL/6J hybrid genetic background were used for all experiments with phosphomutant mice. For reason of clarity in the manuscript we report first the experiments on phosphomutant and then those on CD1 outbred mice. All of the experiments were performed blinded to the genotype and/or the treatment. All procedures related to animal care and treatments conformed to the guidelines and policies of the European Communities Council, and the Animal Care and Use Committee of John Hopkins University, and were approved by the Italian Ministry of Health. Mice were housed in groups of 3–5 subjects, except during the food deprivation procedure when they were individually housed.

### Behavioral procedures

#### Radial maze task

**Fig. 1:**
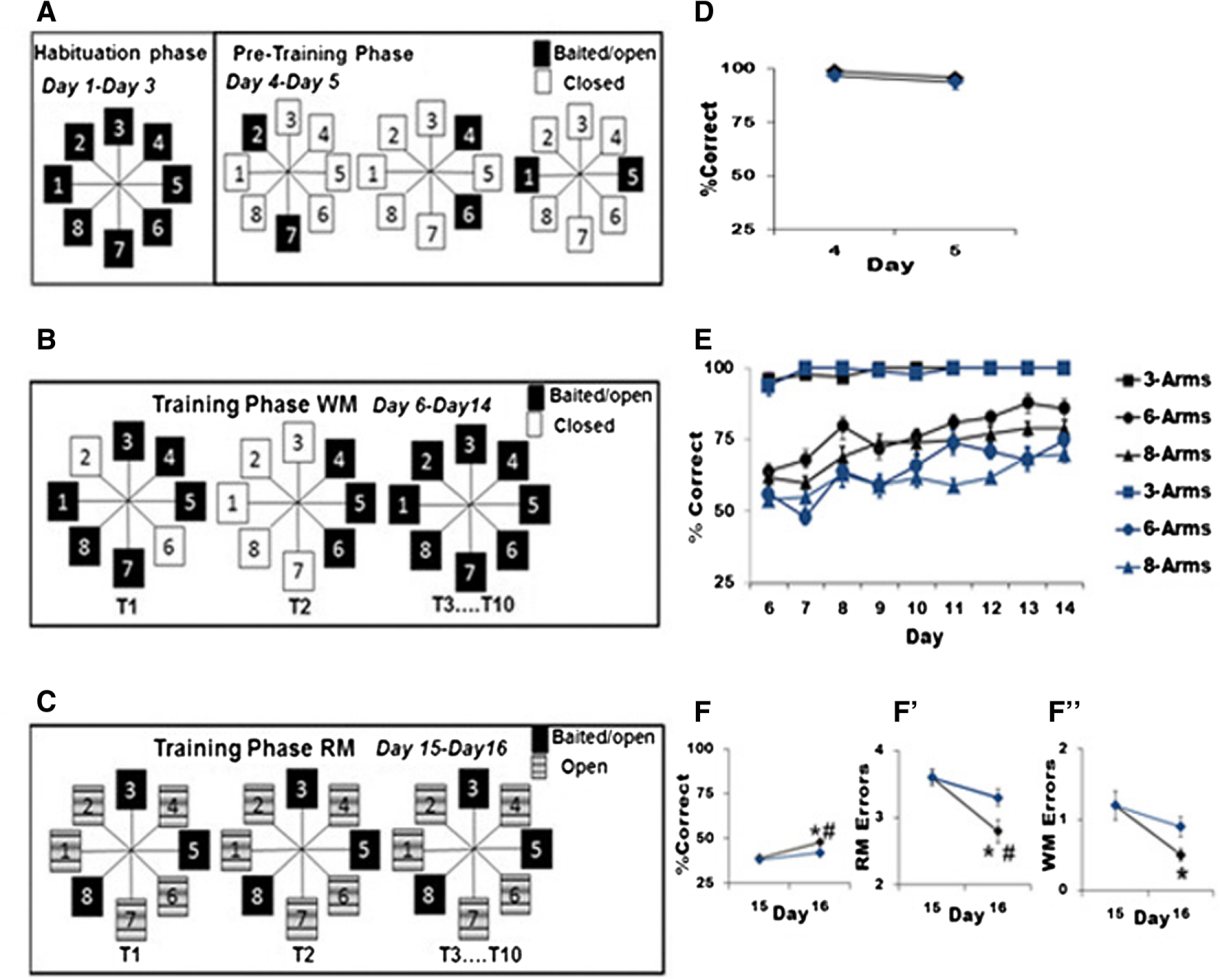
AMPA receptor phosphorylation mediates *spatial* working memory capacity. **a**–**c** Schematic representation of the radial maze procedure used to assess spatial working memory load capacity, and reference memory. **a** The habituation phase lasts 10 min each day for 3 days and allows the animals to explore the apparatus. **a**, **b** During the pre-training and the working memory (*WM*) training phases only baited arms are open. Baited arms were in random position across trials, and never systematically in adjacent positions. In the pre-training phase only two arms are baited/open, the other 6 are closed (**a**). In the WM training phase we modified the memory load by increasing the number of open/baited arms, randomly switching between 3, 6 and 8 open arms within session (**b**). **c** The last 2 days a reference memory (*RM*) training phases was performed, in which all arms were open, but only 3 of them were baited. The 3 baited arms remained constant between trials and days. **d**, **e** Phospho-free (*blue lines*) mice made the same % of correct responses as WT in trials with only 2 open arms during pre-training (**d**) or 3 open arms during training (*squares*) (**e**). In contrast, they were impaired when they had to retrieve food from 6 or 8 open arms (*circles* and *triangles*) (**e**). **f**, **f**″ Phospho-free mice showed lower % of correct responses (**f**), higher mean number of RM (**f**′) and WM (**f’**′) errors compared to WT (*black lines*) during the reference memory phase. *p < 0.05 day 16 vs. day 15 within genotype; ^#^ *p* < 0.05 phospho-free vs. WT, within day, Duncan post hoc tests

Phospho-free and WT littermates were subjected to *the radial maze task,* between 15 and 30 days after being subjected to the DOT–IOT (see below). The apparatus consisted of eight equally spaced arms (Med Associates Inc.) radiating from a small circular, central platform, which were baited at the end with food pellets. The central hub (MED Associates, ENV-338V) consisted of a white polypropylene octagonal base (21 cm in diameter, 7 cm sides). The arms radiated from the center hub with equal spacing between each arm. Each arm was 37 cm long and 7 cm wide with clear polycarbonate walls (13 cm high), and covered with strips of polycarbonate of the same length. The central hub was equipped with eight guillotine doors (MED Associates, ENV-339U). A food trough (ENV-303W) was placed at the end of each arm. A filled 20-mg pellet dispenser (ENV-203-20) was placed behind each food trough. The entire maze was elevated 37 cm above the floor and placed in a well-lit room. During the test period, the body weight of each animal was maintained between 83 and 85 % of the original weight. The 4-phased procedures are reported in Fig. 1a–c. During the habituation phase (Day 1–3) the animals were allowed to explore the apparatus for a 10-min trial each day for 3 days (Fig. 1a). The pre-training phase (Day 4–5) consisted of 10 training trials per day for 2 days with only two baited open arms; the other 6 arms were closed (Fig. 1a). Baited arms were different between trials within the same day, and also within trials between days, and between trials they generated different configurations with adjacent or non/adjacent baited arms to avoid the systematic use of egocentric strategies. The WM load was then manipulated by modifying the number of baited/open arms. The WM training phase (Day 6–14) consisted of 10 training trials per day for 9 days. The procedure was basically the same as the one used in the pre-training phase except that the trials varied from 3, 6 and 8 baited/open arms: 3–4 trials with 3 baited arms (the other 5 closed), 3–4 trials with 6 baited arms (the other 2 closed), and 3–4 trials with 8 baited arms (Fig. 1b). The order of the trials and the choice of the baited arms were random within day and between days. The number of entries and order of entrance was recorded. A WM error was codified as re-entering in an open arm already visited previously. We did not limit the use of the sequential strategy (entering arms in a sequential order), but we evaluated whether the animals relied on it, by counting the number of consecutive arms entered each trial. Data were analyzed by calculating the percentage of sequential (sequential/total × 100). The last phase, called reference memory training (Day 15–16), consisted of 10 training trials for two additional consecutive days (Fig. 1c). This phase differed from the WM training phase in two aspects: all eight arms were always open and the baited arms were kept constant across trials and across days, so that animals could rely on reference memory (RM) to select them. We recorded the number of entries and order of entrance. An RM error was codified as entering in an unbaited arm. The WM errors (number of re-entries in an already visited arm) were calculated, and then divided into RM errors and WM errors.

#### Different object task/identical object task (DOT–IOT)

**Fig. 2:**
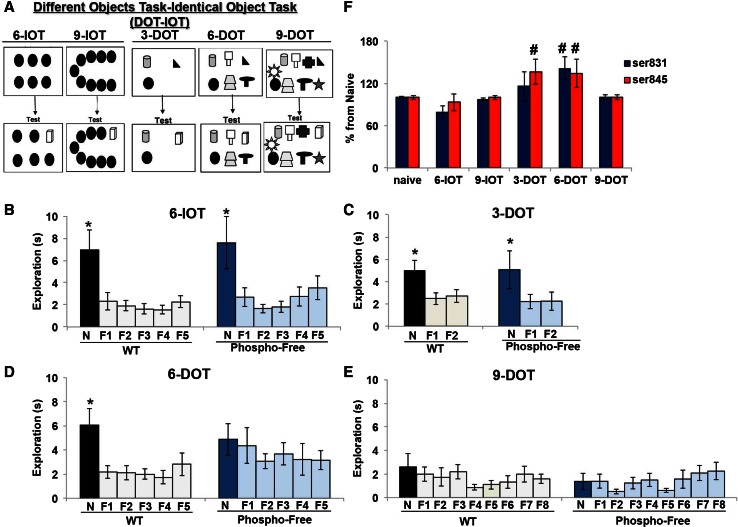
AMPA receptor phosphorylation mediates *object* memory capacity. **a** Schematic representation of the study and test phase of the object memory span task at 1-min RTI. **b**–**e** Performance of phospho-free and WT mice in the DOT–IOT task at 1-min RTI. Phospho-free mice, as well as WT animals, explored the new object (*N*) significantly more than all familiar objects (*F*) in low memory load condition (6-IOT and 3-DOT) (**b**, **c**). In high memory load condition (6-DOT) different from WT, they did not prefer the new object (**d**). Both WT and phospho-free mice did not prefer the new object in the 9-DOT (**e**). **f** Histograms report percentage increase (above the naïve group) of GluA1 AMPA-R phosphorylation at S831 and S845, measured by western blot, in naïve animals and animals exposed to 6 identical (6-IOT), 9 identical (9-IOT), 3 different (3-DOT), 6 different (6-DOT) or 9 different (9-DOT) objects. The results show that AMPA-R phosphorylation was significantly increased at both serine sites after the exposure to 6 different objects (6-DOT), and at S845 after the 3-DOT. *p < 0.05 new (*N*) vs. all the other familiar (*F*) objects, Duncan post hoc tests. ^#^ *p* < 0.05 different vs. naïve group, Student’s *t* test

The complete standard procedure of the different object task/identical object task (DOT–IOT) was thoroughly described in a previous study (Sannino et al. 2012). The DOT–IOT enables the study of MC in rodents by modifying the classical version of the object recognition task (ORT) (Sannino et al. 2012). In the ORT, rodents are firstly exposed to two identical objects and subsequently tested on their ability to discriminate between a familiar and a new object. Since the memory load of this task is very low (1 object), it was augmented by increasing the stimulus set size, i.e., the number of different objects (DOT) (3, 4, etc.) (Fig. 2a). In the control task, the Identical-Objects-Task (IOT), the number of identical objects was increased, without increasing the amount of information to be processed (Fig. 2a). On the testing day, animals were isolated for 15 min in a waiting cage, and then subjected to the habituation trial (10 min) in the empty test cage (35 × 47 × 60 cm); after 1 min inter-trial interval (ITI) animals were submitted to the study phase, during which they were allowed to explore the objects until they had accumulated 35 s of total objects exploration for the identical object task (IOT), or when 5 min elapsed. The maximal area occupied by each object was about 49 cm^2^. For the different objects task (DOT), the study phase was terminated when the animals had accumulated 105, 210, 315 s of total objects exploration for the 3, 6 and 9 different objects tasks, respectively. A maximum of 10 or 15 min was given in the 3, 6 and 9 different objects, respectively, to collect the mentioned exploration times. If the animals did not explore for more than 5 s they were excluded from the test. After an RTI of 1 min for the short-term memory test, and 24-h RTI for the long-term memory test, animals were exposed to identical copies of the familiar objects and one novel object, randomly replacing one of the familiar objects. Animals’ behavior was recorded for 5 min by a video- tracking system (Any-maze, Stoelting, USA) and analyzed by trained observers. This procedure initially developed for CD1 outbred mice was slightly adapted for the C57BL/6J background (that of the phospho-free mice), which needed a week of pre-test habituation (Online Resource 1, Supplementary methods). At the end of the DOT–IOT, a sub-group of these animals (11 WT and 9 phospho-free) was used for the radial maze.

#### AMPA receptors phosphorylation at S845 and S831

Mice were killed 1 min after being subjected to the study phase, by cervical dislocation and their whole brains were rapidly removed. The dorsal hippocampus was punched out. The samples were homogenized in RB buffer (in mM): 10 Na phosphate, pH 7.0, 100 NaCl, 10 Na pyrophosphate, 50 NaF, 1 Na orthovanadate, 5 EDTA, 5 EGTA, 1 µM okadaic acid, and 10 U/ml aprotinin. The homogenates were then centrifuged at 12,000 RCF at 4 °C for 5 min, and the crude membrane pellets were re-suspended in RB buffer. Protein concentration was determined by the BCA protein assay (Pierce, Rockford, IL); about 30 µg of proteins per lane were loaded onto a pre-cast SDS-polyacrylamide gel and analyzed by western blotting with antibodies specific for GluA1 S831 (Upstate Biotechnology, Lake Placid, NY, #PC246, RRID: AB_564636) or S845 (Invitrogen, Italy, #368300, RRID: AB_431401), and actin (Sigma, Italy, #A1978, RRID: AB_476692) (protein loading control). Immunoreactivity was detected by chemiluminescence. Specific bands on chemiluminescence films were quantified by densitometry using the ImageJ gel Software (RRDI: nif-0000-30467). Data are expressed as percentage from naïve (1-week handled mice) or from vehicle-injected animals.

#### Surgery

Cannula implantation and injection procedures were identical to that previously described (De Leonibus et al. 2005). The stereotaxic coordinates used were AP = −1.9 mm; *L* = −1.5 mm, DV = −0.8 mm relative to bregma, according to the atlas of Franklin and Paxinos (1997). Only animals with correct injector placements, verified under a light microscope by analyzing consecutive coronal brain sections (50 μm) stained with Cresyl Violet, were included in the statistical analysis.

#### Drugs

0.1 mg/ml autocamtide-2-related inhibitory peptide (AIP), and 0.05 µg/0.3 µl Rp-adenosine 3′,5′-cyclic monophosphorothioatetriethylammonium salt hydrate (Rpc) were dissolved in PBS; 8 mg/ml anisomycin (Aniso) was dissolved in 60 % distilled water and 40 % DMSO (dimethyl sulfoxide); 2.5 mg/ml dl-2-amino-5-phosphonovaleric acid (AP-5), 2 mg/ml [2.3-dihydroxy-6-nitro-7-sulfamoyl-benzo (F) quinoxaline] (NBQX) were dissolved in distilled water; Avertin solution: 2,2,2-tribromoethanol was dissolved in 5 mL 2-methyl-2-butanol and 200 mL distilled water. All drugs were purchased from Sigma (Italy) and injected in a quantity of 0.3 µl/side. All drug doses were chosen on the basis of the current literature (Tinsley et al. 2009; Vianna et al. 2003). Pre-training and post-training injection protocols were used to analyze the effects of the drugs on WM and LTM, respectively (see Supplementary methods reported in Online Resource 1 for a detailed description of the procedure).

### Statistical analysis

All data are expressed as mean ± SEM. Radial maze: two-way ANOVA (genotype: WT and phospho-free) for repeated measures was used to analyze the % correct response (number of correct arm entries/number of total entries × 100) during pre-training (2 days). During training a three-way ANOVA (genotype: WT and phospho-free) for repeated measures was used to analyze the % correct response during the 9 training days, considering trials with 3, 6 and 8 arms. We further compared the two groups by comparing only trials with 6 and 8 arms, because the lack of error in the 3 arms precluded this analysis. Statistical significance was set at *p* < 0.05. DOT–IOT: ANOVA for repeated measures was used to test the effects of treatments (ex. drug effects, or genotype) as between group variables and objects (different levels: 3, 6, 9). Novel object discrimination, independently on the number of familiar objects, was defined as: the new object was explored significantly more than all the other familiar objects based on results of the Duncan post hoc test (for details see (Sannino et al. 2012). Only this within-group criterion allows to detect novel object discrimination and to compare the results of experiments using different numbers of objects we performed. Statistical significance was set at *p* ≤ 0.05; only significant *p* values were reported.

## Results

### GluA1 AMPA receptor phosphorylation mediates memory load capacity

We tested the hypothesis that hippocampal GluA1 AMPA-R phosphorylation regulates MC.

To this aim we utilized the knock-in mutant mice that specifically lack both of S845 and S831 phosphorylation sites on the GluA1 subunit (“phospho-free mice”) (Lee et al. 2003). Phospho-free and WT mice were subjected to a radial arm maze procedure designed to assess the contribution of the information load on spatial memory. The WM load (0 RTI) was increased by increasing the number of open/baited arms (Fig. 1a–c). Phospho-free mice made the same percentage of correct response as controls during the pre-training phase, when they had to retrieve food from only two open arms (Fig. 1d). During training, they were subjected to 10 different trials each day, with a random number of open/baited arms (3, 6 or 8 open) (Fig. 1b). During this phase, several variables had a significant effect on the percentage of correct responses, specifically: genotype (F1/18 = 45.618; *p* < 0.0001), days (F8/144 = 16.967; *p* < 0.0001), number of open arms (F2/36 = 545.493; *p* < 0.0001), the interaction between days × number of open arms (F16/288 = 4.951; *p* < 0.0001) and the genotype × number of open arms (F2/36 = 20.060; *p* < 0.0001) (Fig. 1e). This analysis clearly shows, first of all, that there was a drop of the performance of both groups when 6 or 8 arms were open as compared to only 3 arms. A direct statistical comparison between 6 and 8 arms showed a significant effect of the variables genotype (F1/18 = 40.318; *p* < 0.0001), days (F8/144 = 15.556; *p* < 0.0001), number of open arms (F1/18 = 27.836; *p* < 0.0001), and interaction between days × number of open arms × genotype (F8/144 = 2.354; *p* < 0.02). These results indicate that by increasing the number of open arms from 6 to 8, we further increased the memory load and that phospho-free mice were selectively impaired compared to WT littermates when they had to retrieve food from 6 and 8 open arms, but not from 3 open arms (Fig. 1e). Post hoc analysis (not reported in the figure) showed a significant improvement in performance across training days (from day 6 to day 14) only in the WT group in the 6 open arms condition (*p* = 0.01). This improvement in the WT group might be due to processes not related to WM. Indeed, repeating the task across days might have reduced the WM load by favoring long-term storage of novel spatial information constant across the task, such as the identity of the spatial cues and their spatial configuration. This might have also favored GluA1 protein synthesis in both genotypes, compensating for the phosphorylation defect. Furthermore, overtraining across days might have promoted the acquisition of non-spatial strategies to solve the task, as suggested by the significant increase in the use of sequential strategy (Fig. S1 reported in Online Resource 1). Nevertheless, differently from the 3-arm version of the task, performance never reached 100 % correct in the 6- and 8-arm versions. This suggests that all the mentioned factors are not sufficient to eliminate: (1) the WM component of the task and (2) the memory load difference between the different conditions (3, 6, and 8 open arms). Moreover, these factors do not seem to account for the different performance between WT and phospho-free mice. Indeed, the two genotypes did not differ in the acquisition of the sequential strategy (Fig. S1 reported in Online Resource 1), or in the time to complete the test [(F8/144 = 37.292; *p* < 0.0001), arms (F2/144 = 495.148; *p* < 0.0001); days × arms (D16/288 = 11.282; *p* < 0.0001)] (data not shown). Nevertheless, their performance (% correct) was significantly different all over the training period: on days 8, 10, 12, 13 (*p* < 0.0001; *p* = 0.004; *p* = 0.01; *p* = 0.01, respectively) with 6 open arms, and on all training days, except day 7 (*p* < 0.02; *p* = 0.006; *p* = 0.04; *p* = 0.005; *p* < 0.0001; *p* = 0.02; *p* = 0.008; *p* = 0.02) with 8 open arms, suggesting that there was a further WM load-dependent defect between 6 and 8 arms in the phospho-free group.

Interestingly, when tested in the spatial version of the water maze task, phospho-free mice have been shown to perform normally at 0, but not at 24-h RTI (Lee et al. 2003), as compared to WT control animals, which suggested that AMPA-R phosphorylation is necessary only for LTM and not for acquisition in this task. Therefore, we subjected the same mice to an additional radial maze procedure requiring a combination of both reference (at 24-h RTI) and WM. In this aim, all eight arms were opened, but only 3 of them were baited. In this way, mice were required to rely on both RM, as the position of baited arms was kept constant between trials and on WM for remembering which of the arms was already visited within the same trial. To confirm that phospho-free mice are also impaired in reference memory we baited 3 out of 8 arms, as we showed that they were not impaired when retrieving food from 3 baited arms in the WM version of the task (Fig. 1e). Phospho-free mice as compared to WT animals, not only showed lower percentage of correct responses, on the second training day [genotype (F1/18 = 3.89; *p* = 0.06); Days (F1/18 = 23.743; *p* = 0.0001); genotype × day (F1/18 = 23.743; *p* = 0.0001)] (Fig. 1f), but they also showed higher number of RM errors [days (F1/18 = 28.828; *p* < 0.0001); genotype × day (F1/18 = 5.22; *p* = 0.03)] (Fig. 1f′). Furthermore, although we did not find a significant effect of the variable genotype or of the interaction between genotype and days, but only a significant effect of the latter [days (F1/18 = 9.012; *p* = 0.007)], a deeper inspection of the data suggested that phospho-free mice made significantly higher number of WM errors as compared to WT on the second testing day (Fig. 1f″). The fact that phospho-free mice were impaired in retrieving food from 3 baited arms only when they had to be selected among 8 open arms further confirms that their behavior is sensitive to the memory load and that the defect observed in the different memory load conditions is not due to the differing amount of reward gained when 3, 6 or 8 arms are open. All together these findings suggest that AMPA-R phosphorylation *is necessary* for both spatial WM and LTM in conditions of high load.

Spatial memory by definition is based on the formation of associative memory; this makes it extremely difficult to manipulate the information load in spatial memory tasks, as it is difficult to control the number of multiple associations the subject relies on. Therefore, we subjected phospho-free and WT mice to the DOT–IOT (see Online Resource 1, Supplementary methods), in which we modulated the memory load by increasing the number of different objects during the study phase (Fig. 2a). At 1-min RTI, phospho-free mice, as well as controls, performed normally in the 6-IOT (Fig. 2b) (low memory load), showing no significant impairment in the 3-DOT (intermediate memory load) (Fig. 2c). In the 6-DOT (Fig. 2d) (high memory load), the post hoc analysis clearly demonstrated that they did not discriminate the new as compared to the entire familiar ones. In the 9-DOT, as expected based on our previous findings (Sannino et al. 2012), both WT and phospho-free mice were impaired (Fig. 2e). Phospho-free mice did not differ from WT for total object exploration time during the study phase (Table 1). All together these data show that phospho-free mice do not have a general impairment in object memory at short RTI, but *they do have reduced object MC*.

**Table 1:** Object exploration during the study phase of phospho-free mice

Experiment	Genotype	TMOE (s)	*N*
3 DOT	WT	32 ± 5	13
	Phospho-free	31 ± 7	13
6 IOT	WT	22 ± 4	13
	Phospho-free	27 ± 2	9
6 DOT	WT	56 ± 5	11
	Phospho-free	61 ± 9	10
9 DOT	WT	61 ± 15	9
	Phospho-free	57 ± 13	11

Total mean object exploration (TMOE) ± SEM during the study phase in the DOT–IOT of WT and phospho-free mice. The table reports also the number (*N*) of subjects for each group. There are no significant differences between WT and phospho-free

To address whether the stimulus set size regulates AMPA-R phosphorylation within the hippocampus different groups of adult naïve mice were exposed to an increasing number of different objects (3, 6 and 9), or to identical objects (6 and 9) as a control (Fig. 2a), and after 1 min they were killed for measuring tissue GluA1 AMPA-R phosphorylation at both S845 and S831 within the hippocampus by western blot analysis and compared with naïve animals. We found that AMPA-R phosphorylation (S845: F5/67 = 2.72; *p* = 0.02; S831: F5/67 = 4.094; *p* = 0.002) is dependent on the stimulus set size (Fig. 2f; Fig. S2 reported in Online Resource 1). We observed no changes in AMPA-R phosphorylation in a low load condition (6-IOT), a selective increase at S845 after exposure to an intermediate load (3-DOT), and a significant increase at both serine sites only in a high memory load condition (6-DOT). Interestingly, in animals exposed to the 9-DOT, the overload condition associated with impaired discrimination of the new object (Fig. 2e) we did not detect any change in AMPA-R phosphorylation, as compared to the 9-IOT control (Fig. 2f; Online Resource 1). Consistent with previous findings, during the study phase of the 9-DOT mice explored significantly more (F4/50 = 50.03; *p* < 0.0001) than in all other conditions (3-DOT, 6-DOT– and IOT) (Table 2). The lack of increased phosphorylation in the 9-DOT is not the result of a compensatory increase in total GluA1 levels (Naïve = 100 ± 2.16; 9-IOT = 104 ± 5; 9-DOT = 94 ± 3; *p* = n.s.). This suggests that AMPA-R phosphorylation is specifically correlated with the active processing of object-related information.

**Table 2:** Object exploration during the study phase of naïve CD1 mice subjected to the DOT–IOT, and then used for western blot experiment (referred to Fig. 2)

Task (number of subjects)	CD1 naïve
6-IOT (*N* = 12)	34 ± 1.2
9-IOT (*N* = 9)	33 ± 1.4
3-DOT (*N* = 10)	80 ± 13*
6-DOT (*N* = 12)	161 ± 14*
9-DOT(*N* = 12)	212 ± 16*

Total mean object exploration (TMOE) ± SEM during the study phase in the DOT-IOT of CD1 mice. The table reports also the number (*N*) of subjects for each group. *p < *0.05* vs. all the other groups

All together these data clearly show that AMPA-R phosphorylation is regulated by information load, and that this process is necessary for memory load capacity at short RTI, providing the first identified molecular mechanism associated with *memory capacity limit*.

### Molecular pathways that regulate MC in high memory load conditions within the dorsal hippocampus

AMPA-R phosphorylation at S831 and S845 is promoted by NMDA-CaMKII and PKA, respectively. When activated, these kinases phosphorylate not only AMPA-Rs, which we showed to be necessary for memory at short RTI, but also the cyclic AMP response element-binding protein phosphorylation (Abel et al. 1997), thereby promoting novel protein synthesis known to be necessary for LTM. This suggests that the activation of the NMDA/AMPA-protein kinases might be a common pathway to support memory in conditions of high load, independently of the RTI.

**Fig. 3:**
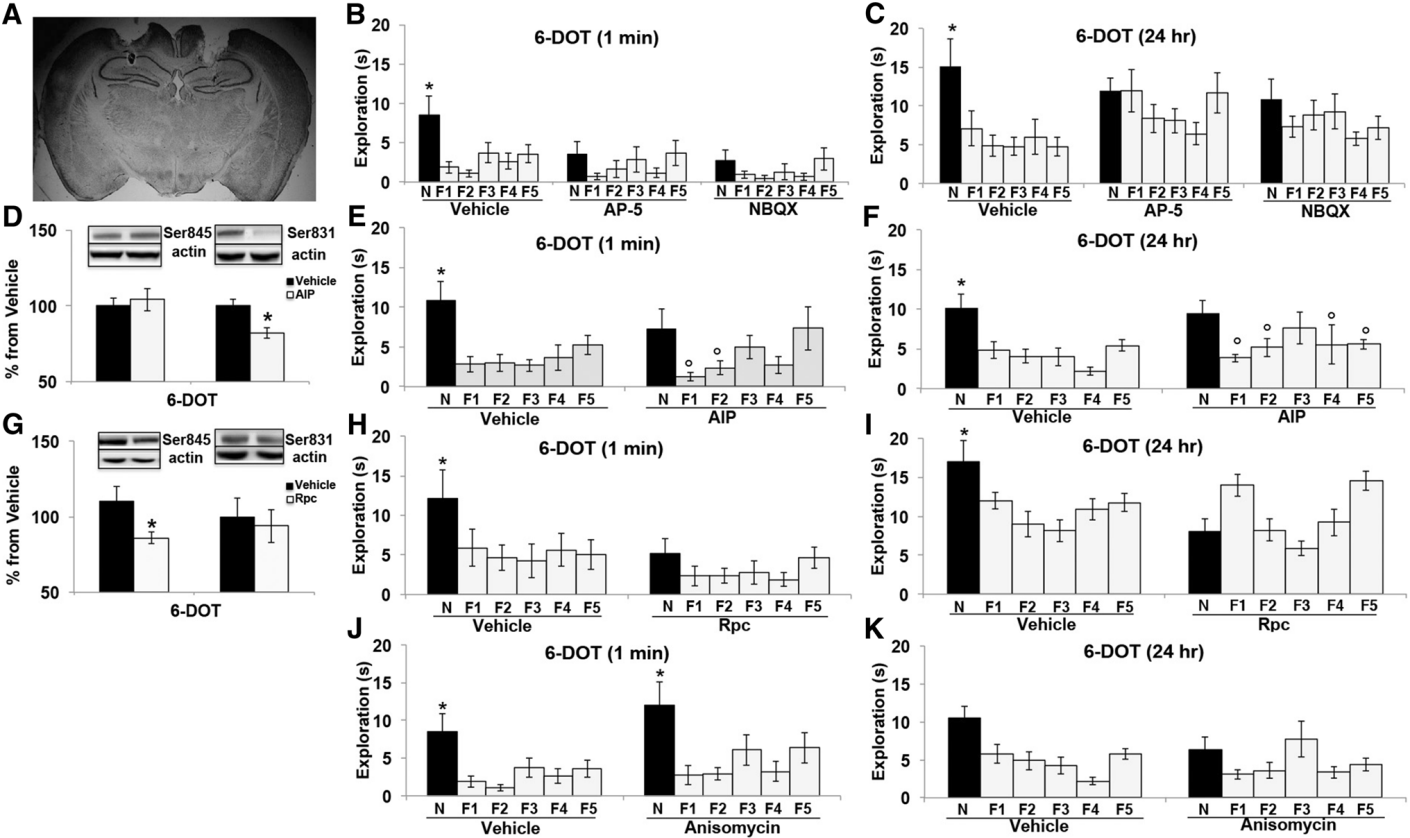
In high memory load condition object LTM shares the same molecular pathway as object memory at short retention interval with the addition of novel protein synthesis. **a** Photos of representative Nissl-stained coronal sections of animals bilaterally implanted with permanent cannulae and injected within the hippocampus. **b**, **c**, **e**, **f**, **h**, **i**, **j**, k Effects of hippocampus focal pre-training and post-training administrations of inhibitors on the preference for the new object (*N*) and compared to familiar objects (*F*) in the test phase of the 6-DOT performed at 1-min and 24-h RTI, respectively. In all cases vehicle-injected animals explored the new object significantly more than all the other familiar objects. Pre-study phase injection of AP-5 (**b**), NBQX (**b**), AIP (**e**), and Rpc (**h**) but not of anisomycin (**j**) impaired the preference for the new object as compared to the familiar object at 1-min RTI. With AIP, however, this effect was partial, as the new object was still preferred as compared to 2 of the familiar objects. Pre-study phase injection of AIP (**d**) and Rpc (**g**), as compared to injection of vehicle, significantly reduced the percentage increase (above the vehicle group) of AMPA-R phosphorylation at S831 and S845, respectively, induced 1 min after the exposure to 6 different objects. Post-study phase administration of all treatments fully impaired performance in the 6-DOT at 24-h RTI (**c**, **i**, **k**), except for the AIP (**f**), where the new object was preferred against 4 (out of 5) of the familiar objects. *p < 0.05 new vs. all the other familiar objects, within treatment. *p < 0.05 new (*N*) vs. all the other familiar (F) objects; °*p* < 0.05 new (*N*) vs. each the other familiar (F) objects, Duncan post hoc tests

**Fig. 4:**
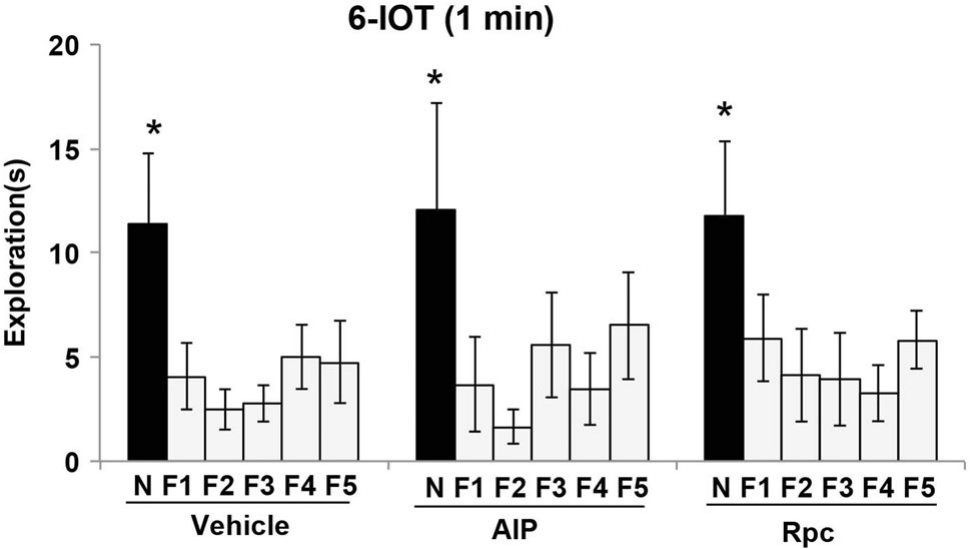
Kinase inhibitors injected within the hippocampus do not affect object memory at short retention interval in low memory load condition. Pre-training phase administration of CaMKII (*AIP*) and PKA (*Rpc*) inhibitors at the same dose that has been shown to affect performance in the 6-DOT does not affect new object preference in the 6-IOT. *p < 0.05 new (*N*) vs. all the other familiar (*F*) objects

To test this hypothesis, we used a pharmacological approach. Using bilateral cannula permanently implanted (Fig. 3a) below the dorsal hippocampus, we focally injected selective inhibitors. We performed pre-training injection and testing at 1-min RTI, and post-training injection and testing at 24-h RTI, to study the effects of memory acquisition and consolidation, respectively. This study was performed using the 6-DOT, as it allows pre-training and post-training pharmacological manipulation. The results show that all vehicle-treated groups explored the new object significantly more than the entire set of familiar objects at both 1-min and 24-h RTI, suggesting that in a high memory load condition they were able to perform the task independent of the RTI. Focal administration of the NMDA and the AMPA/kainite receptor antagonist, AP-5 and NBQX, respectively, impaired performance in the 6-DOT at both 1-min (Fig. 3b) and 24-h (Fig. 3c) RTI. To test whether CaMKII and PKA mediate both the increase in AMPA-R phosphorylation and memory maintenance in the high memory load task, we focally injected the CaMKII inhibitor, AIP, or the PKA selective inhibitor, Rpc. After the study phase, half of the animals were killed to measure AMPA-R phosphorylation at S845 and S831, while the other half was subjected to the test phase to assess memory. Pharmacological blockade of CaMKII (Fig. 3d) or PKA (Fig. 3g) during the pre-study phase significantly reduced AMPA-R phosphorylation induced by the exposure to 6 different objects at the S831 and S845, respectively, as compared to vehicle-treated animals. As expected, this effect was highly specific for their respective phosphorylation sites (Fig. 3d, g), and no effect was observed on total GluA1 (data not shown). The blockade of both kinases impaired new object discrimination. Indeed, pre-training administration of AIP significantly impaired new object discrimination as compared to 3 of the 5 familiar objects (Fig. 3e). A complete lack of object discrimination was observed in the group treated with the PKA inhibitor, Rpc (Fig. 3h). No significant effects were observed on total object exploration during the study phase (Table 3), or when the same drugs were injected before the 6-IOT (Fig. 4). In LTM experiments, post-training CaMKII blockade slightly impaired new object discrimination 24 h later (Fig. 3f), since the new object was explored significantly more than all the other 4 familiar objects (F1, F2, F4, for F5), except one (F3). The comparison between the effect of the same dose of AIP at 1-min (Fig. 3e) and 24-h (Fig. 4f) RTI seems to suggest that, it was more effective on short than on long RTI. In contrast, animals injected with the PKA inhibitor, Rpc, were completely impaired in discriminating the new object as compared to the familiar objects (Fig. 3i).

**Table 3:** Object exploration during the study phase of mice subjected to the DOT–DOT–IOT in the behavioral pharmacological experiments (referred to Fig. 3)

TR	RTI	Drug	TMOE	*N*
Pre-training	1 min	Vehicle	108 ± 13	15
		AP-5	98 ± 17	7
		NBQX	84 ± 9	7
		Vehicle	111 ± 14	13
		AIP	108 ± 10	7
		Vehicle	169 ± 12	8
		Rpc	147 ± 20	8
		Vehicle	108 ± 13	15
		Aniso	128 ± 21	7
Post-training	24 h	Vehicle	132 ± 13	9
		AP-5	134 ± 18	9
		NBQX	95 ± 15	8
		Vehicle	95 ± 7	12
		AIP	91 ± 16	8
		Vehicle	170 ± 12	8
		Rpc	185 ± 9	9
		Vehicle	101 ± 12	12
		Aniso	80 ± 12	10

Total mean object exploration (TMOE) ± SEM during the study phase in the DOT–IOT of groups treated with vehicle and inhibitors, 15 min before the habituation phase and tested at 1-min retention time interval (RTI), and in groups treated immediately after the study phase and tested at 24-h RTI. The table reports also the number (*N*) of subjects for each group. There are no significant differences between inhibitors and vehicle.TR: treatment protocol

These results provide the first behavioral pharmacological evidence demonstrating that both PKA and CaMKII activity within the dorsal hippocampus can affect object memory under high memory load conditions, and the associated AMPA-R phosphorylation.

The activation of these kinases has been suggested to mediate LTM by promoting the synthesis of new proteins. We therefore tested whether memory performance under conditions of high memory load was dependent on the synthesis of de novo proteins. As expected, we found that pre-study phase administration of anisomycin did not affect object exploration during the study (Table 3) and the test phase at short RTI (Fig. 3j). In contrast, when anisomycin was injected immediately after the study phase, and the animals were tested 24 h later, a clear lack of new object discrimination was observed (Fig. 3k). This suggests that novel protein synthesis within the dorsal hippocampus is required in high memory load conditions, only when LTM has to be formed.

## Discussion

In this study, we provide insights into understanding of the neurobiology of MC. We demonstrated that knock-in mice with prevented phosphorylation of AMPA-Rs at the two key residues, S845 and S831, have reduced object and spatial WM capacity, as well as reduced spatial LTM. Then we report that memory load regulates AMPA-R phosphorylation within the hippocampus, and that MC limit is paralleled by a lack of AMPA-R phosphorylation. Both MC and AMPA-R phosphorylation are reduced by the pharmacological inhibition of CaMKII and PKA pathways within the hippocampus, specifically in conditions of high load and short RTI. Even though the stabilization of this information into LTM does activate the same pathways, it additionally requires de novo synthesis of proteins. To our knowledge, this is the first molecular mechanism that has been suggested to support the processing of increasing memory load.

### Hippocampal AMPA receptor phosphorylation mediates memory load capacity

It has been previously shown that the hippocampus is involved in regulating memory capacity. However, to our knowledge, no study has addressed the molecular mechanisms activated within the hippocampus to process high information load. A central role of hippocampal AMPA-R in short-term memory has been recently suggested. Using knock-out GluA1 mice it has been shown that GluA1-containing AMPA receptors are involved in short-term habituation, and in modulating the intensity or perceived salience of stimuli (ex. the novel object) (Sanderson et al. 2009, 2010). The effect of AMPA-R blockade on novel object discrimination at short-delay we report in this study is line with these previous findings. In this study we show, however, that this effect is dependent on the number of items presented to the animals. Moreover, we show that an additional post-translational plastic mechanism of GluA1 AMPA-R is necessary for short-term memory in conditions of high memory load, which is AMPA-R phosphorylation at S845 and S831. Phosphorylation at these two sites has been observed in several brain regions, including the hippocampus, immediately after exposure to a variety of stimuli (Bevilaqua et al. 2005; Whitlock et al. 2006; Havekes et al. 2007; Cui et al. 2012). Here, we provide the first evidence that AMPA-R phosphorylation is specifically regulated by information load. By showing that an overloaded condition (9 different objects), which is associated with a lack of new object discrimination and a lack of increase in AMPA-R phosphorylation, we provide the first experimental evidence of a possible molecular mechanism underlying MC limit.

Except for few studies (Ferretti et al. 2014, Lee et al. 2003), in the earlier published data it was generally not demonstrated whether behavioral experience-dependent increase in AMPA-R phosphorylation was necessary for memory acquisition. Using mice with knock-in mutations in the GluA1 phosphorylation sites (“phospho-free mice”) (Lee et al. 2003), we could demonstrate that a lack of AMPA-R phosphorylation at S845 and S831 reduced object memory capacity from 6 to 4, establishing in this way *a causal link* between AMPA-R phosphorylation and memory load capacity. We also report here for the first time that the spatial WM deficit in phospho-free mice is memory load dependent. These mice are not impaired when they have to retrieve food from 2 or 3 arms, but show a clear impairment when 6 or 8 arms are open.

The identified role of AMPA-R phosphorylation in supporting high memory load is consistent with electrophysiological findings showing that AMPA-R phosphorylation is a rapidly induced plasticity mechanism (occurring in a few minutes). Furthermore, this is consistent with its role in increasing synaptic currents generated by the release of glutamate from axon terminals in vitro (Barria et al. 1997a, b, Derkach et al. 1999, Banke et al. 2000), and in general with its ability to potentiate AMPA function.

### The CaMKII–PKA pathway within the hippocampus controls AMPA-R phosphorylation and memory performance in high memory load conditions

Consistently, with the well-known role of glutamate gated ion channels, NMDA-R and AMPA-R, in hippocampal synaptic plasticity and memory formation, here we show that they are also involved in object memory in conditions of high load, independent of the RTI. This result is in apparent contradiction with findings showing that GluA1 KO mice show enhanced spatial memory at long RTI (Sanderson et al. 2009). The different training conditions (single trial vs. repeated), as well as the different memory load demand (high vs. low) of the tasks used in the two studies can account for the different results obtained.

We also show here that at short RTI, the activation of CaMKII and PKA is necessary for novel object discrimination in high memory load conditions, and for high memory load-induced increase in AMPA-R phosphorylation. PKA, and to a lesser degree CaMKII, activation is also necessary for object LTM. There is a wealth of data on the role of CaMKII and PKA activation within the hippocampus, which is a crucial step for LTM formation and in particular in spatial memory (Silva et al. 1992a, b; Abel et al. 1997; Da Silva et al. 2013). Interestingly, in the case of low memory load (2 equal objects task) injection of the CaMKII inhibitor, AIP, into the perirhinal cortex does not affect immediate memory (0 RTI), but only LTM (Tinsley et al. 2009); our results in the 6-IOT are consistent with these prior findings. Our findings in high memory load conditions are consistent with the suggested crucial role of CaMKII in short-term potentiation, and of PKA in both short- and long-term plasticity (Xie et al. 2008; Wang et al. 2008; Silva et al. 1996; Barsegyan et al. 2010; Michel et al. 2008; Irvine et al. 2005). Interestingly, the evidenced role of dopamine D1 receptors in modulating WM load in the frontostriatal circuit might also be exerted through the activation of PKA (Aultman and Moghaddam 2001; Backman et al. 2011; Cools et al. 2008; Snyder et al. 1998). This suggests that common molecular mechanisms might be concurrently activated in the frontostriatal and mediotemporal circuits in high memory load conditions.

By demonstrating that PKA in the hippocampus is involved at both short and long RTI under certain conditions we challenge the classical dualistic temporal distinction of memory (Dash et al. 2007; Shrager et al. 2008; Sanderson et al. 2009), and demonstrate that the two processes can share not only the same neural pathway (the hippocampus) but also the same molecular mechanisms (PKA and AMPA-R phosphorylation). Nevertheless, the dissociation of the effects of anisomycin at 1-min and 24-h RTI suggests that this common pathway dissociates into two separate processes at the molecular level that leads to AMPA-R phosphorylation within few minutes and to novel protein synthesis within hours, to support memory at short- and long-RTI, respectively.

## Conclusions

The data we present in this study fill an important gap in our knowledge on the molecular substrates of memory capacity. Our data expand on previous findings (Wan et al. 1999; Zhu et al. 1996; Sannino et al. 2012) showing that when the subject is confronted with one or a few objects (low memory load: 6-IOT/3-DOT), object-related information does not recruit the hippocampus into the task. In contrast, being confronted with many different objects (high memory load) might generate highly convergent inputs that are able to recruit the hippocampus, and once there they trigger NMDA-R activation, which leads to postsynaptic calcium influx that in turn activates CaMKII/PKA pathways, as previously suggested (Cammarota et al. 2004). These protein kinases once activated can exert bidirectional control over bottom-up, such as AMPA surface receptors phosphorylation, and top-down, such as novel protein synthesis, biological processes necessary for memory at short- and long-RTI, respectively.

This suggests the intriguing hypothesis that information loading may *drive cooperation* between otherwise competing or parallel processes (WM vs. LTM), representations (spatial vs. item), neural circuits (hippocampus vs. cortex), and molecular pathways (kinase activation vs. kinase inhibition). We speculate that *AMPA*-*R phosphorylation might be the site of this cooperation*: it might support rapid learning in conditions of high memory load by favoring synaptic currents generated by release of glutamate from axon terminals (Barria et al. 1997a, b; Derkach et al. 1999; Banke et al. 2000),while at the same time directly influencing long-term synaptic changes by favoring the stabilization of GluA1 subunit at the plasma membrane (He et al. 2009; Ferretti et al. 2014). This latter process has been shown to lead to an increase of calcium permeable AMPA receptors (CP-AMPA) (He et al. 2009), which might further promote calcium-sensitive enzyme activation, generating a “*reverberating*” positive loop favoring the transition from WM to LTM.

## Electronic supplementary material

Below is the link to the electronic supplementary material.

ESM1 (DOCX 150 kb)

## References

[CR1] Abel T, Nguyen PV, Barad M, Deuel TA, Kandel ER, Bourtchouladze R (1997). Genetic demonstration of a role for PKA in the late phase of LTP and in hippocampus-based long-term memory. Cell.

[CR2] Aultman JM, Moghaddam B (2001). Distinct contributions of glutamate and dopamine receptors to temporal aspects of rodent working memory using a clinically relevant task. Psychopharmacology.

[CR3] Backman L, Karlsson S, Fischer H, Karlsson P, Brehmer Y, Rieckmann A, Macdonald SW, Farde L, Nyberg L (2011) Dopamine D(1) receptors and age differences in brain activation during working memory. Neurobiol Aging 32(10):1849–1856 10.1016/j.neurobiolaging.2009.10.018 19962789

[CR4] Banke TG, Bowie D, Lee H, Huganir RL, Schousboe A, Traynelis SF (2000). Control of GluR1 AMPA receptor function by cAMP-dependent protein kinase. J Neurosci.

[CR5] Barria A, Derkach V, Soderling T (1997). Identification of the Ca2+/calmodulin-dependent protein kinase II regulatory phosphorylation site in the alpha-amino-3-hydroxyl-5-methyl-4-isoxazole-propionate-type glutamate receptor. J Biol Chem.

[CR6] Barria A, Muller D, Derkach V, Griffith LC, Soderling TR (1997). Regulatory phosphorylation of AMPA-type glutamate receptors by CaM-KII during long-term potentiation. Science.

[CR7] Barsegyan A, Mackenzie SM, Kurose BD, McGaugh JL, Roozendaal B (2010). Glucocorticoids in the prefrontal cortex enhance memory consolidation and impair working memory by a common neural mechanism. Proc Natl Acad Sci USA.

[CR8] Beason-Held LL, Rosene DL, Killiany RJ, Moss MB (1999). Hippocampal formation lesions produce memory impairment in the rhesus monkey. Hippocampus.

[CR9] Benke TA, Luthi A, Isaac JT, Collingridge GL (1998). Modulation of AMPA receptor unitary conductance by synaptic activity. Nature.

[CR10] Bevilaqua LR, Medina JH, Izquierdo I, Cammarota M (2005). Memory consolidation induces *N*-methyl-d-aspartic acid-receptor- and Ca2+/calmodulin-dependent protein kinase II-dependent modifications in alpha-amino-3-hydroxy-5-methylisoxazole-4-propionic acid receptor properties. Neuroscience.

[CR11] Cammarota M, Bevilaqua LR, Bonini JS, Rossatto JI, Medina JH, Izquierdo N (2004). Hippocampal glutamate receptors in fear memory consolidation. Neurotox Res.

[CR12] Cools R, Gibbs SE, Miyakawa A, Jagust W, D’Esposito M (2008). Working memory capacity predicts dopamine synthesis capacity in the human striatum. J Neurosci.

[CR13] Cui B, Wu M, She X, Liu H (2012). Impulse noise exposure in rats causes cognitive deficits and changes in hippocampal neurotransmitter signaling and tau phosphorylation. Brain Res.

[CR14] Da Silva WC, Cardoso G, Bonini JS, Benetti F, Izquierdo I (2013). Memory reconsolidation and its maintenance depend on L-voltage-dependent calcium channels and CaMKII functions regulating protein turnover in the hippocampus. Proc Natl Acad Sci USA.

[CR15] Dash PK, Moore AN, Kobori N, Runyan JD (2007). Molecular activity underlying working memory. Learn Mem.

[CR16] De Leonibus E, Oliverio A, Mele A (2005). A study on the role of the dorsal striatum and the nucleus accumbens in allocentric and egocentric spatial memory consolidation. Learn Mem.

[CR17] Derkach V, Barria A, Soderling TR (1999). Ca2+/calmodulin-kinase II enhances channel conductance of alpha-amino-3-hydroxy-5-methyl-4-isoxazolepropionate type glutamate receptors. Proc Natl Acad Sci USA.

[CR18] Engle RW, Tuholski SW, Laughlin JE, Conway AR (1999). Working memory, short-term memory, and general fluid intelligence: a latent-variable approach. J Exp Psychol Gen.

[CR19] Ferretti V, Perri V, Cristofoli A, Vetere G, Fragapane P, Oliverio A, Ammassari-Teule M, Mele A (2014). Phosphorylation of S845 GluA1 AMPA receptors modulates spatial memory and structural plasticity in the ventral striatum. Brain Struct Funct.

[CR20] Franklin K, Paxinos G (1997). The mouse brain in stereotaxic coordinates.

[CR21] Havekes R, Timmer M, Van der Zee EA (2007). Regional differences in hippocampal PKA immunoreactivity after training and reversal training in a spatial Y-maze task. Hippocampus.

[CR22] Hayashi Y, Shi SH, Esteban JA, Piccini A, Poncer JC, Malinow R (2000). Driving AMPA receptors into synapses by LTP and CaMKII: requirement for GluR1 and PDZ domain interaction. Science.

[CR23] He K, Song L, Cummings LW, Goldman J, Huganir RL, Lee HK (2009). Stabilization of Ca2+-permeable AMPA receptors at perisynaptic sites by GluR1-S845 phosphorylation. Proc Natl Acad Sci USA.

[CR24] Irvine EE, Vernon J, Giese KP (2005). AlphaCaMKII autophosphorylation contributes to rapid learning but is not necessary for memory. Nat Neurosci.

[CR25] Klostermann EC, Braskie MN, Landau SM, O'Neil JP, Jagust WJ (2012) Dopamine and frontostriatal networks in cognitive aging. Neurobiol Aging 33(3):623.e15–623.e24. doi:10.1016/j.neurobiolaging.2011.03.002 10.1016/j.neurobiolaging.2011.03.002 PMC3245323 21511369

[CR26] Lee HK, Takamiya K, Han JS, Man H, Kim CH, Rumbaugh G, Yu S, Ding L, He C, Petralia RS, Wenthold RJ, Gallagher M, Huganir RL (2003). Phosphorylation of the AMPA receptor GluR1 subunit is required for synaptic plasticity and retention of spatial memory. Cell.

[CR27] Levy DA, Hopkins RO, Squire LR (2004). Impaired odor recognition memory in patients with hippocampal lesions. Learn Mem.

[CR28] Michel M, Kemenes I, Muller U, Kemenes G (2008). Different phases of long-term memory require distinct temporal patterns of PKA activity after single-trial classical conditioning. Learn Mem.

[CR29] Reisel D, Bannerman DM, Schmitt WB, Deacon RM, Flint J, Borchardt T, Seeburg PH, Rawlins JN (2002). Spatial memory dissociations in mice lacking GluR1. Nat Neurosci.

[CR30] Sanderson DJ, Good MA, Skelton K, Sprengel R, Seeburg PH, Rawlins JN, Bannerman DM (2009). Enhanced long-term and impaired short-term spatial memory in GluA1 AMPA receptor subunit knockout mice: evidence for a dual-process memory model. Learn Mem.

[CR31] Sanderson DJ, McHugh SB, Good MA, Sprengel R, Seeburg PH, Rawlins JN, Bannerman DM (2010). Spatial working memory deficits in GluA1 AMPA receptor subunit knockout mice reflect impaired short-term habituation: evidence for Wagner’s dual-process memory model. Neuropsychologia.

[CR32] Sannino S, Russo F, Torromino G, Pendolino V, Calabresi P, De Leonibus E (2012). Role of the dorsal hippocampus in object memory load. Learn Mem.

[CR33] Saunders NL, Summers MJ (2010). Attention and working memory deficits in mild cognitive impairment. J Clin Exp Neuropsychol.

[CR34] Shi SH, Hayashi Y, Petralia RS, Zaman SH, Wenthold RJ, Svoboda K, Malinow R (1999). Rapid spine delivery and redistribution of AMPA receptors after synaptic NMDA receptor activation. Science.

[CR35] Shi S, Hayashi Y, Esteban JA, Malinow R (2001). Subunit-specific rules governing AMPA receptor trafficking to synapses in hippocampal pyramidal neurons. Cell.

[CR36] Shrager Y, Levy DA, Hopkins RO, Squire LR (2008). Working memory and the organization of brain systems. J Neurosci.

[CR37] Silva AJ, Paylor R, Wehner JM, Tonegawa S (1992). Impaired spatial learning in alpha-calcium-calmodulin kinase II mutant mice. Science.

[CR38] Silva AJ, Stevens CF, Tonegawa S, Wang Y (1992). Deficient hippocampal long-term potentiation in alpha-calcium-calmodulin kinase II mutant mice. Science.

[CR39] Silva AJ, Rosahl TW, Chapman PF, Marowitz Z, Friedman E, Frankland PW, Cestari V, Cioffi D, Sudhof TC, Bourtchuladze R (1996). Impaired learning in mice with abnormal short-lived plasticity. Curr Biol.

[CR40] Snyder GL, Fienberg AA, Huganir RL, Greengard P (1998). A dopamine/D1 receptor/protein kinase A/dopamine- and cAMP-regulated phosphoprotein (Mr 32 kDa)/protein phosphatase-1 pathway regulates dephosphorylation of the NMDA receptor. J Neurosci.

[CR41] Tarantino IS, Sharp RF, Geyer MA, Meves JM, Young JW (2011). Working memory span capacity improved by a D2 but not D1 receptor family agonist. Behav Brain Res.

[CR42] Tinsley CJ, Narduzzo KE, Ho JW, Barker GR, Brown MW, Warburton EC (2009). A role for calcium-calmodulin-dependent protein kinase II in the consolidation of visual object recognition memory. Eur J Neurosci.

[CR43] Vianna MR, Igaz LM, Coitinho AS, Medina JH, Izquierdo I (2003). Memory extinction requires gene expression in rat hippocampus. Neurobiol Learn Mem.

[CR44] Wan H, Aggleton JP, Brown MW (1999). Different contributions of the hippocampus and perirhinal cortex to recognition memory. J Neurosci.

[CR45] Wang H, Feng R, Phillip Wang L, Li F, Cao X, Tsien JZ (2008). CaMKII activation state underlies synaptic labile phase of LTP and short-term memory formation. Curr Biol.

[CR46] Whitlock JR, Heynen AJ, Shuler MG, Bear MF (2006). Learning induces long-term potentiation in the hippocampus. Science.

[CR47] Xie W, Ramakrishna N, Wieraszko A, Hwang YW (2008). Promotion of neuronal plasticity by (−)-epigallocatechin-3-gallate. Neurochem Res.

[CR48] Young JW, Meves JM, Tarantino IS, Caldwell S, Geyer MA (2011). Delayed procedural learning in alpha7-nicotinic acetylcholine receptor knockout mice. Genes Brain Behav.

[CR49] Zamanillo D, Sprengel R, Hvalby O, Jensen V, Burnashev N, Rozov A, Kaiser KM, Koster HJ, Borchardt T, Worley P, Lubke J, Frotscher M, Kelly PH, Sommer B, Andersen P, Seeburg PH, Sakmann B (1999). Importance of AMPA receptors for hippocampal synaptic plasticity but not for spatial learning. Science.

[CR50] Zhu XO, McCabe BJ, Aggleton JP, Brown MW (1996). Mapping visual recognition memory through expression of the immediate early gene c-fos. NeuroReport.

