# Supplementary material for: Phosphorylation of the AMPA receptor GluA1 subunit regulates memory load capacity
Source: Brain Struct Funct. 2014 Nov 8;221(1):591–603. doi: 10.1007/s00429-014-0927-1 (PMC4425615; doi:10.1007/s00429-014-0927-1)
Supplement: Supplementary file 1 — ESM1 (DOCX 150 kb) [file 429_2014_927_MOESM1_ESM.docx]

**ELECTRONIC SUPPLEMENTARY MATERIAL**

**Title: Phosphorylation of the AMPA receptor GluA1 subunit regulates memory load capacity**

**Laura Olivito^1^, Paola Saccone^2^, Valentina Perri^3-4^, Julia L. Bachman^5^, Paola Fragapane^6^, Andrea Mele^3-4^, Richard L. Huganir^5*^, Elvira De Leonibus ^1-2*^**

1. Institute of Genetics and Biophysics, CNR, Naples, Italy
2. Telethon Institute of Genetics and Medicine (TIGEM), Naples, Italy
3. Dipartimento di Biologia e Biotecnologie, Università degli Studi di Roma “La Sapienza”, Rome, Italy
4. Centro di Ricerca in Neurobiologia-D. Bovet, Università degli Studi di Roma “La Sapienza”, Rome, Italy
5. Department of Neuroscience and Howard Hughes Medical Institute, Johns Hopkins University School of Medicine, Hunterian 1001, 725 North Wolfe Street, Baltimore, MD 21205, USA
6. Istituto di Biologia e Patologia Molecolare, CNR, Rome, Italy

**Corresponding authors:**

Elvira De Leonibus, Institute of Genetics and Biophysics, CNR, Via P. Castellino 111, 80131 Naples, Italy, Tel: +39-081-7473433, Fax:+39-081-7472037 e-mail: [deleonibus@igb.cnr.it](mailto:deleonibus@igb.cnr.it)

*Identical contribution to the study

**Online Resource 1**

**Supplementary methods**

***Behavioral procedure to adapt the DOT-IOT task to the C57BL/6J:***

The week before testing C57BL/6J phospho-free mice and their relative WT control mice, were habituated to the testing room for about one hour each day and then placed in transparent cages (24 x 18 x 13.5 cm) for 10 min. The experimenter handled them and introduced small objects into the cage (i.e. pencil, small cups). Furthermore, the three-object task was performed using a smaller test cage (16 x 16 x 12 cm) for these mice, as they did not pay attention to the objects in the standard-sized test cage. Each animal was subjected 2 or 3 times to the object task, at a distance of at least 15 days. The test order was randomized between the different stimulus set-sizes, and each time a different set of objects was used.

The C57Bl/6J mouse strain is much less explorative than the CD1 outbred line, as evidenced by the results reported in Tables 1 and 2 and in the figures 1 and 2 for the test phase. Nevertheless, C57Bl/6J normal mice solve the task as well as CD1 outbred mice: (i) they do prefer the novel object as compared to the familiar once in low memory load conditions (6-IOT, 3-DOT), can discriminate up to 6 different objects (6-DOT), but not 9 different objects (9-DOT). These results are not consistent with some previous findings reported an impaired capacity of C57Bl/6J to perform the standard version of the object recognition task ([Orsini et al. 2004](#_ENREF_1)). Nevertheless, they are consistent with plenty of publications showing that as well as CD1 mice they can perform this task ([Porton et al. 2010](#_ENREF_2); [Repetto et al. 2014](#_ENREF_3)).

***Pre-training and post-training injection protocols.***

To study the effects of these drugs at short RTI they were injected 10-15 min before the 10 min habituation. After 1 min animals were then subjected to the study phase, and after another 1 min RTI they were subjected to the test phase. We refer to this injection protocol as “pre-training” injection in the text. To study the effects of these drugs in LTM they were injected immediately after the study phase. Animals were left in their waiting cages for an additional hour and then returned to their home cage. The test phase was performed 24 hours later. We refer to this injection protocol as “post-training” injection in the text. Using a post-training administration procedure allowed us to conclude that the effects observed on LTM were not secondary to eventual deficits occurring at short RTI, because the animals were off-drug during the study phase.

**Supplementary Figures**

**
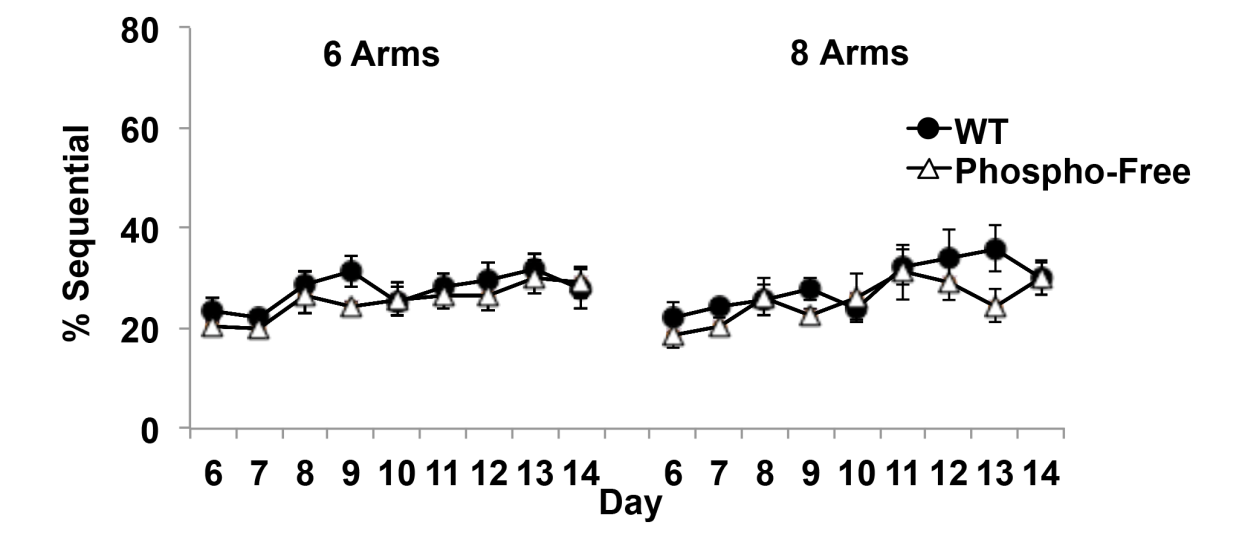
**

**Fig S1 Analysis of sequential strategy use in the radial maze.** The figure reports the percentage of consecutive (sequential) arm entries in the wild-type and phospho-free mice during training in the radial maze with 6 or 8 arms open. Both groups similarly increased the percentage of sequential arms entries across days [6 arms (F8/144=3.06; p=0.003); 8 arms (F8/144=3.177; p=0.002). No significant effect of genotype or of the interaction between genotype and days was found. This clearly suggests that any improvement in WM performance across days might be in part due to the development of this strategy, and that the learning defect observed in phospho-free mice was not due to impairment in egocentric (sequential) learning.





**Fig S2** **Representative protein bands on chemiluminescence films.** Representative bands obtained by subjecting different groups of mice to different number of identical (6-IOT, 9-IOT) or different objects (3-DOT, 6-DOT, 9-DOT), compared with the naïve groups. The western blot experiments were performed firstly on naïve, 6-IOT, 6-DOT and 3-DOT groups (**a**); another batch of animals was added for measuring protein GluR1 S845 and S831 after the animals were subjected to the 9-DOT and 9-IOT and compared with an additional naïve group (**b**). Densitometry quantification of the bands are expressed as percentage from the naïve group and reported in Fig. 2F.

**References**

Orsini C, Buchini F, Conversi D, Cabib S (2004) Selective improvement of strain-dependent performances of cognitive tasks by food restriction. Neurobiology of learning and memory 81 (1):96-99

Porton B, Rodriguiz RM, Phillips LE, Gilbert JWt, Feng J, Greengard P, Kao HT, Wetsel WC (2010) Mice lacking synapsin III show abnormalities in explicit memory and conditioned fear. Genes, brain, and behavior 9 (3):257-268. doi:10.1111/j.1601-183X.2009.00555.x

Repetto D, Camera P, Melani R, Morello N, Russo I, Calcagno E, Tomasoni R, Bianchi F, Berto G, Giustetto M, Berardi N, Pizzorusso T, Matteoli M, Di Stefano P, Missler M, Turco E, Di Cunto F, Defilippi P (2014) p140Cap regulates memory and synaptic plasticity through Src-mediated and citron-N-mediated actin reorganization. The Journal of neuroscience : the official journal of the Society for Neuroscience 34 (4):1542-1553. doi:10.1523/JNEUROSCI.2341-13.2014
